# Leadership in informal stormwater governance networks

**DOI:** 10.1371/journal.pone.0222434

**Published:** 2019-10-17

**Authors:** Brian C. Chaffin, Theresa M. Floyd, Sandra L. Albro

**Affiliations:** 1 W.A. Franke College of Forestry & Conservation, University of Montana, Missoula, MT, United States of America; 2 College of Business, University of Montana, Missoula, MT, United States of America; 3 Holden Forests & Gardens, Cleveland, OH, United States of America; University of Vermont, UNITED STATES

## Abstract

Recent transitions in the governance of urban stormwater, specifically developments that leverage the environmental and social benefits of green infrastructure (GI) including infiltration and neighborhood stabilization, often require capacities beyond those of any single municipal- or regional-scale organization. In many cities, transitions toward green stormwater infrastructure have been shepherded by networks of individuals spanning a diversity of organizations from governments to NGOs. These networks are often informal, that is, not established by legal mandate, governing authority, or formal agreement, and are often striking for their lack of formal hierarchy or formal leadership. Previous scholarship has revealed the importance of leadership in the development and efficacy of these multiorganizational, cross-sector environmental governance networks, but research has yet to empirically investigate and characterize informal network leaders within the context of GI for stormwater mitigation. To address this gap, we designed and administered a social network analysis (SNA) survey to individuals in a regional network of GI stormwater management professionals in and around Cleveland, Ohio USA. We collected network data on individual relationships, including collaboration and trust, and tested the impact of these relationships on peer-recognition of leaders in the GI network. Our findings suggest that network size, frequency of collaboration, and individual position within the network—specifically, betweenness centrality and openness—defined and likely supported leaders in the stormwater governance network. Leaders in this non-hierarchical, multi-institution context were more likely to be women and brokerage roles within the network benefitted women, not men, which contrasts with previous findings from research on single-organization and corporate networks. The implications of this research suggest that informal environmental governance networks, such as the GI network investigated, differ substantially from the generally more hierarchical networks of organizations. This finding is useful for municipalities and regional authorities grappling with complex environmental challenges, including transitions in strategies to manage excess stormwater for the protection of municipal drinking water sources and urban freshwater ecosystems.

## Introduction

A growing body of scholarship on environmental governance has identified charismatic leaders with diverse skill sets and high social and political capital as an essential ingredient for transitioning informal networks of governance actors past intractable conflict and toward governance approaches that recognize myriad competing human values, the inherent uncertainty of global change, and the complexity of social-ecological systems [1–4]. Contexts of high uncertainty and complexity define modern environmental governance challenges; such is the case of urban stormwater management, as aging infrastructure and dramatic increases in impervious surfaces fail to keep pace with a changing climate and changes in the frequency and intensity of precipitation patterns [5,6]. Although previous research has demonstrated that informal, cross-sector networks are critical to successfully addressing complex environmental governance challenges such as those posed by urban stormwater, we know very little about the leaders that guide these networks [7]. To date, research on leadership in environmental governance generally, and on informal network governance specifically, has relied strongly upon anecdotal evidence and narratives of change, and lacks a robust body of empirical analyses aimed at better understanding network leaders and the contexts through which they emerge. Further, recent reviews have revealed that ‘leaders’ and ‘leadership’ are often assumed to represent an unequivocal and uncontested good across environmental science and environmental governance literatures, thus meriting a more critical analysis [8,9].

In response, this paper presents research designed to empirically investigate peer-nominated leaders in an informal, cross-sector, multi-institution network of stormwater management professionals working collectively toward adoption of green infrastructure (GI) to mitigate and manage urban stormwater in Cleveland, Ohio, USA [10]. We consider this network “informal” because: it is not officially sanctioned by any government, statute, charter, agreement (e.g. memorandum of understanding or MOU), or legal charge; it is not hierarchical; and all members participate on a voluntary basis. Further, “leaders” in this network are not designated as such through formal role, title, or position of authority, but are instead identified by other individuals as inhabiting a leadership role within a diffuse network of actors representing multiple private organizations and public-sector agencies [11–13]. Although the Northeast Ohio Regional Sewer District (NEORSD), which has a service area that includes the city of Cleveland, is under a court-enforced consent decree which requires that a percentage of stormwater abatement is achieved via GI approaches [14], an informal network of GI professionals has emerged outside of this legal framework to increase knowledge, implementation, and acceptance of GI strategies for stormwater management in the region [10,15]. We asked this informal network of GI professionals who they identified as leaders critical for the success of GI projects and then examined: (1) personal characteristics of these leaders, including gender, role, and organizational affiliation; (2) the characteristics of leaders’ individual networks; and (3) how specific network characteristics differentially affect individuals’ likelihood of leadership nomination by their peers. Through these tests, we build a more nuanced understanding of how informal network leadership in this context differs from that of other sectors, such as in networks defined by organizational membership that have frequently been studied in the business sector. This research has the potential to aid informal networks of environmental governance actors in fostering leadership and leadership capacity, and increasing the speed and efficacy with which they can mobilize resources, achieve goals, and navigate the complexity and uncertainty of modern environmental challenges.

## Background

### Leadership in environmental governance networks

Over the past several decades, a substantial body of literature has demonstrated that cross-sectoral, multidisciplinary, and multiorganizational networks of actors play critical roles in environmental governance [16–22]. Many of these networks are informal and organic, not officially sanctioned, mandated, or chartered by any government or hierarchical entity, and they frequently emerge in response to a need or pressing resource conflict [23]. Networks fill a gap between the formal processes of government and organizations and the more informal social processes that also influence environmental governance outcomes [7]. In this context, environmental governance is defined as the collective processes through which society: (1) negotiates values surrounding the use and conservation of natural resources; (2) establishes goals for environmental management based on the outcomes of this negotiation; and (3) implements actions to achieve these goals [24–27]. The influence and effectiveness of networks in environmental governance is in part based on the structure of networks themselves. Researchers have theorized that *bonding ties* within a network—collaboration or communication relationships between network actors—enable individuals to collaborate more effectively, while *bridging ties*—relationships between network actors and influential outsiders—allow well-connected network actors to access outside resources and influence [17,20,28–31]. For example, strong bonding—measured as network closure—has been found to be associated with adaptability [17,32], greater ability to solve collaboration problems between disparate actors [33,34], and participation in management initiatives [35], while networks characterized by actors who are bridges between diverse groups have been found to be associated with greater engagement by stakeholders in water management activities [20], the development of stronger ties between stakeholders and greater trust in the fairness of policy [21], and the use of sustainable practices [36]. Additionally, networks characterized by centralized structures (those in which a core group of central actors are closely connected, and peripheral network members are primarily connected to the core group rather than to each other) have been found to be associated with more effective cooperation [19], collective action [37], and maintaining agreed-upon protocols for effective communication [38].

Leadership is oft cited as unequivocally important for building successful and effective environmental governance networks for mitigating and/or adapting to various environmental ‘crises’ such as the negative societal consequences of climate change, the overuse and degradation of water resources, rapid rates of biodiversity loss, and the tensions between conservation and development [1,3,8]. Leadership has been defined primarily in research contexts outside of the environmental sciences as an individual’s ability to influence groups of people to achieve common goals [39–41]. In line with this view, peer-reviewed literature in environmental science reveals a distinct positive bias toward individual leadership [42] and largely forwards normative assumptions that leadership and individual characteristics or traits of leaders such as charisma, strength, commitment, or perseverance are inherently positive, good, and desirable qualities with positive influence on environmental outcomes [9]. Individual leaders are referred to as entrepreneurs, champions, brokers, or change makers, and the absence of these individuals is strongly connected to reported negative or ineffective outcomes in environmental governance [43–46]. A recent review of leadership in the environmental sciences literature by Evans et al. [9] and expanded by Case et al. [8] demonstrates that the majority of studies promoting these normative assumptions are more descriptive than empirical and fail to adequately interrogate leadership beyond the scale of individual characteristics inherent to the ‘chosen few.’ Evans et al. [9] report the idea “…that ‘we all know what leadership is’ appears to be taken for granted, which reduces it to a term of lay convenience rather than one of robust social scientific validity.” The authors go on to point out that a few studies do attempt to disaggregate leadership from outcomes [47,48], but many instead employ additional proxies to measure outcomes, further blurring any potential correlations with leadership.

Some empirical studies, however, do explore specific aspects of leadership in environmental governance, including: the negative impacts of leadership; the relationship between leader and followers; the relationship between leadership and scale; and leadership as a relational process beyond individuals [8,9,49]. It is this last aspect we explore in this work. Theories on distributed leadership, shared leadership, and emergent leadership developed primarily in business scholarship, and specifically in organizational contexts, have reconceptualized leadership as a collective social process that emerges through the interactions of a group of actors, i.e. a network [11,12,50–53], thus moving beyond trait and behavior theories focused on the individual leader. Leadership depends not only on the leader, but also on followers’ recognition and acceptance of leadership [41]. Leadership that emerges through the interactions of a group of actors (“emergent leadership”), in particular, can be thought of as either: (1) awarded to an actor or actors because of achievements such as contribution to the goals of the group or group functional performance; or (2) ascribed to an actor or actors because of individual characteristics that fit with the perceivers’ ideas of a ‘leader’ [54]. Ascribed leadership has often been found to be over-awarded to men, due to the ‘think leader, think male’ paradigm described in social role theory and leader categorization theory [55–57]; however, contextual conditions within groups have been found to weaken this paradigm, even showing that in certain circumstances, women, rather than men, are more likely to emerge as leaders [58–60].

Leadership is inseparable from the historical, cultural, political, and *environmental* contexts that manifest the need for leaders and leadership to arise [61–65]. Thus, applying theories of emergent leadership—developed primarily through research within the business context—to informal environmental governance networks is potentially problematic because the contextual differences between organizational network and environmental governance network situations are numerous and significant. As one example, organizational employees have, by virtue of their membership in the organization, agreed to exert effort towards achieving their organization’s goals [66]. In contrast, stakeholders in an environmental governance or resource-use network may have many different organizational affiliations (including government, NGO, direct resource user, etc.), and thus potentially divergent—even competing—goals and priorities. As Bodin and Prell [7] state: “The very nature of the … resource governance context is typically very different from what characterizes a corporation” (369). As another example, the largely volunteer nature of members’ participation in environmental governance networks might make communal leadership behaviors more effective for influencing others to achieve goals, which might enhance women’s opportunities to be perceived as leaders within this context [55,67]. Hence, the opportunity exists to expand empirical testing of leadership, including interactions with gender, within the context of larger-scale, multi-organization environmental governance networks. Accordingly, we apply network theory and social network analysis to better understand the influence of network structure and individual network position on peer-identified leaders within an informal environmental governance network.

### Informal network leadership

Informal network leadership has been a topic of interest in social network research almost from its inception, in part because social networks themselves are often seen as informal alternatives to the more formal, generally hierarchical structure of organizations [68]. Thus, informal network leadership was conceived of as the power and influence accorded to individuals due to their structural position within the informal network, rather than from formal title or role within an organizational hierarchy [69]. Carter et al. [61] expanded this definition to describe leadership to also include “…informal processes existing in parallel to, or in place of, formal hierarchical structures” (599). Environmental governance networks are generally informal, operating through organic, self-regulated processes, and made up of interested individuals from a variety of groups such as governments, government agencies, environmental/conservation NGOs, and other citizen-based organizations who recognize that a heightened opportunity for organizational goal attainment is associated with participation in an external network [70]. Individuals who serve as informal leaders within these environmental governance networks may or may not be formal leaders in their respective organizations (e.g. hold a formal title or role), but they are ascribed leadership status by others in the network due to a combination of their performance, demonstration of skills useful for navigating diffuse, multiorganizational networks, and their fit with others’ perceptions of a leader (which could be both related to their individual traits such as gender, and/or their organizational affiliations [71,72]). Thus, leaders in informal environmental governance networks can be conceived as “… emergent rather than predefined and that their role can only be understood through examining the relationships within the group (rather than by focussing [sic.] on his/her personal characteristics or traits)” (17) [41]. We explicitly investigate this phenomenon of leadership herein, where our emphasis on ‘informal’ is situated more on the network itself and less on the style or process of leadership exhibited by individuals.

Given the varying contexts (historical, political, cultural) through which informal environmental governance networks arise, there is no formula for leadership and no ideal type for the informal network leader [41,73]. Instead, informal leadership can be conceptualized as fluid and an emergent property of the network [74]. In their comprehensive review of leadership theories, Bolden et al. [41] further emphasize the disconnection between informal leaders and formal title or role, highlighting the “…importance of social relations in the leadership contract, the need for a leader to be accepted by their followers and a realisation [sic.] that no one individual is the ideal leader in all circumstances…” (17). Early studies of informal network leadership centered on what Silvia and McGuire [72] refer to as the ‘master list’ approach: identifying individual traits and characteristics useful to predict informal leaders. This approach, however, ignored situational contexts, and gave way to research on behaviors as a way to better understand and predict leadership. In their study of over four hundred public sector emergency managers both within single-agency contexts and multiorganizational network settings, Silvia and McGuire [72] empirically compared differences in leadership behaviors within an individual’s more formal, hierarchical organization and while acting as a participant in an informal, multiorganizational network of other emergency managers. They found that in informal networks ‘people-oriented behaviors’ exceeded both in use and importance the more ‘task-oriented behaviors’ central in a single-agency leadership setting [72]. People-oriented behaviors critical to informal network leaders included information sharing, building and sustaining personal relationships, negotiation and brokering, strategic visioning, and mobilizing resources toward goal achievement; often these behaviors were accompanied by individualized approaches to empathy, active listening, conflict resolution, trust building, creativity, and innovation [72,75,76]. This behavioral approach to informal network leadership research remains dominant today, as informal networks often arise from the need for institutional culture change, innovative and creative vision, and dynamic leadership to navigate complex problems which no single organization has the capacity to resolve alone. Role congruity theory may suggest that this behavioral approach may advantage women’s perception as leaders, since communal behaviors such as information sharing and relationship building are aligned with the stereotypical female role [55,77].

### The informal green infrastructure network for stormwater management in Cleveland, OH

In Cleveland, the elevation of GI as a priority in the 2011 Consent Decree inspired the organization of a network of GI professionals to collectively support the required GI project implementation and to further additional GI projects in Cleveland through the development of planning, design, funding, construction, monitoring, and maintenance—this despite the fact that the court order to abate stormwater volumes with GI was only legally binding on the Northeast Ohio Regional Sewer District (NEORSD). Prior to the consent decree, however, stormwater professionals in Cleveland had taken an interest in GI not only for its potential for stormwater abatement, but also for vacant land reuse and to stabilize declining neighborhoods [78,79].

In Cleveland, population loss of 57% over several decades has resulted in demolition of thousands of abandoned structures and creation of 5,800 acres of urban vacant land, approximately 15% of the city’s land area. The Great Recession hit Cleveland particularly hard, resulting in informal designation as “ground zero” of the foreclosure crisis [80]. Ohio land bank legislation in 2008 streamlined the clearing of abandoned houses and, in conjunction with the Hardest Hit Fund in 2010, facilitated faster creation of vacant land. In response, a citywide effort composed of government, nonprofit organizations, academic institutions, and water utilities mobilized to define strategies to put vacant parcels into productive use to stabilize neighborhood decline, resulting in an initial series of 156 pilot projects that included green stormwater infrastructure [78].

Green infrastructure (GI)—including bioswales, rain gardens, green roofs, pervious pavement, and other projects that enhance the infiltration or detention capacity of the urban environment—is rapidly becoming a widely-accepted approach to complement or replace grey infrastructure solutions for managing stormwater in urban areas [81,82]. GI holds the potential for mitigating high volumes of urban stormwater and curbing combined sewer overflow (CSO) events in a more cost-effective and distributed manner than engineering-based, built grey infrastructure [83–86]. Major barriers to the development of GI for stormwater management have been identified (e.g. [87,88]), including: (1) the variability of soil resources and conditions necessary for effective GI [89,90]; (2) difficulties in financing GI when compared to large, public works with proven return-on-investment rates [91]; (3) land ownership and maintenance issues [10,15]; (4) a lack of coordination between government agencies and other groups across the life span of GI design, implementation, and maintenance [14,35]; and (5) cultural differences, including a general ‘grey vs. green divide’ among GI and stormwater management professionals that often places engineering approaches, community development approaches, and research approaches in conflict as opposed to coordinated [10,92]. A rapidly growing body of scholarship reports general enthusiasm and optimism for leveraging GI to manage urban stormwater, particularly for its potential to restore hydro-ecological structures and processes, as well as ecosystem services lost or diminished in urban development [15,81,85]. The complexity and uncertainty of overlying GI approaches on highly-engineered urban stormwater conveyance systems, however, has deterred regional and municipal sewer organizations (authorities) from embracing and championing the approach beyond large, centralized projects to comply with the consent decree, leaving space for the influence of informal networks.

Networks of professionals convened around vacant land use, stormwater, and the consent decree, and eventually these small networks converged to form the collective Cleveland GI network; today, however, there continues to be significant overlap among the GI network and other networks focused more on vacant land use, stormwater management, and urban development [10,93]. Since early efforts to encourage GI in Cleveland, concerns about long-term land use and liability, as well as growing awareness about CSO events, have reduced emphasis on GI as primarily a vacant land reuse strategy. However, there continues to be significant interest within the network in characterizing and maximizing the social and environmental benefits that GI can bring to the city beyond stormwater capture [93]. The network’s approach to GI in Cleveland involves a combination of large, centralized GI built by the regional sewer authority, as well as smaller, distributed projects undertaken by a variety of local entities [94], some of which are also financially supported by the sewer authority. Each organization represented in the Cleveland GI network has a distinct set of goals, objectives, and approaches for GI projects in Cleveland, often competing with those of other organizations, with no formal hierarchy or structure for determining collective outcomes. Thus, the network provides a unique opportunity to study leadership, specifically, the impact of individual network positions, personal traits, and behaviors on informal, peer-recognized positions of leadership.

### Research questions and hypotheses

Within the context of the voluntary network of professionals actively seeking to expand GI for stormwater management in Cleveland, we were interested in building a better understanding of individuals identified by their peers as leaders, including reasons underlying leadership nomination. Previous research suggests that leaders emerge because of individual attributes, behaviors, and fit between individual leadership style, competencies, and the situation [95]. Thus, we expected that individuals who exercised a regulatory role by virtue of their position, and those who were affiliated with organizations leading the change effort would emerge as leaders of GI projects in Cleveland. We build upon previous research by additionally examining network properties associated with informal leadership.

Our central research question is: ***what network qualities characterize peer-nominated leaders in informal*, *multiorganizational networks for stormwater management*?** Previous social network research suggests that power and influence associated with leadership comes from access to information and control over key resources accorded to individuals by virtue of their network structure and position [96–101]. More recently, research on network leadership has focused on the idea that power and influence also come from bricolage benefits of individuals developing translation, collaboration, and integration skills when they interact with different parts of the network [102]. We hypothesized that both are critical to predicting leadership in informal networks such as the stormwater governance network in Cleveland. Guided by this and our central research question, we tested the following specific hypotheses:

H1: ***Individuals’ collaboration network size will be positively related to their influence over others and hence predict their peer-nomination as leaders in the network***.
H2: ***Individuals’ frequency of collaboration will be positively related to their influence over others and hence predict their peer-nomination as leaders in the network***.

Individuals who collaborate with more partners and who collaborate more frequently have increased access to information and resources, making them valuable partners; they have increased visibility within the network; and they have more opportunities to influence others to achieve goals [96,103–106].

H3: ***Individuals with collaboration networks characterized by openness will be perceived as leaders***.
H4: ***Individuals with collaboration networks characterized by high betweenness centrality will be perceived as leaders*.**

These individuals will be seen as more influential because they have greater access to information and resources; greater control over the flows of information or resources within the network; greater opportunities to accrue bricolage benefits, making them valuable partners; and opportunities to influence others across structural boundaries [105–107].

H5: ***Individuals’ trust network size will be positively related to leadership nomination***. Individuals are more likely to be influenced by people they trust; this influence will likely be correlated with peer-nomination as network leaders [108,109].

We also examined a second research question: ***do network characteristics relate differently to male and female leaders*?** Ascribed leadership is often over-awarded to men, likely because of historic, socially-constructed perceptions of a match between ‘male’ and ‘leader’ in the eyes of many perceivers [55,57,60,67]. This may also be the case because of top-down contextual factors [13,110], such as the structural inequalities that have contributed to males historically dominating high-level positions or formal leadership roles in organizations [111]. Recent research, however, has uncovered factors that mitigate or reverse this relationship, including group gender and personality composition [60], interaction between gender and individual personality traits of potential leaders [71], potential leaders’ network centrality and multiplexity of relationships [112], and interaction between gender and network characteristics [71]. A meta-analysis by Eagly and Karau [113] suggests that women may emerge as informal leaders more often than men in networks charged with tasks that involve more complex social relations. Studies also suggest that when situational contexts require some degree of transformative change (deep structural change), the bias toward the perceived emergence of men as informal leaders shifts [114].Thus, although we did not specifically hypothesize the direction of the relationship, we tested whether and how gender moderates the relationships between network characteristics and peer-nominated leadership.

## Methods

To address our research questions, we designed and administered a social network analysis (SNA) survey instrument to individuals identified as part of an informal regional network of GI stormwater management professionals in and around Cleveland. The survey instrument and research protocols were approved by the University of Montana Institutional Review Board. Written informed consent was obtained from each research participant prior to administering the survey instrument detailed herein. The survey was designed to collect information on relationships between GI professionals in order to construct measures of network connectivity and to statistically test relationships between individuals’ network properties and peer-identified leaders within the network. Applying SNA in this manner is rarely achieved in studies of informal environmental governance networks, due in part to: (1) the often-contentious nature of many environmental and natural resource issues and the subsequent unwillingness of individuals to provide network information; (2) the complexity of environmental governance networks and the difficulties in bounding them for network research [7]; and (3) the methodological difficulty of implementing a whole network study and achieving the response rate necessary to accurately capture the network [115].

To identify leaders within the network, we asked survey respondents to identify individuals “most critical for achieving green stormwater infrastructure in Cleveland.” Survey questions that explicitly inquire about leaders and leadership have been shown to be biased toward men and individuals who occupy formal leadership roles (i.e. those with formal roles and titles within their respective organizations and communities) [56,67,113]. In an effort to avoid this bias in our research, we specifically asked survey respondents to identify those individuals “critical” to green infrastructure. We argue that in this research context—non-hierarchal, volunteer, multiorganizational (i.e. informal) networks—leaders are the ones who are “critical” for getting things done. This does not simply imply completion of tasks, it also implies making decisions, influencing others, driving toward collective goals, and achieving measurable outcomes. Investigations that explore informal leadership in networks research often use words other than “leader” to identify informal leaders in a network. For example, Brass [96] identified informal leaders based on others perceiving them as having “influence,” or having “pull, weight, or clout…” (529). Brass and Burkhardt [99] asked respondents to rate their peers on their level of influence: “please indicate how much influence the person you circled has in the everyday activities of this agency” (455–456). Krackhardt [100] identified informal leaders using questions based on two dimensions: “the ability to get things done despite resistance and the ability to influence through personal magnetism (charisma)” (348). In addition, our key informant encouraged us to avoid the term “leader” as it may have been poorly received by our respondents, due to the democratic and collaborative nature of the GI projects. This strong set of evidence led us to ask respondents who was “critical” for GI for stormwater management in Cleveland as a proxy for identifying leaders in this informal network.

### Sample and procedures

We conducted a purposeful snowball sampling procedure [116] as follows: (1) a key informant who was actively engaged in GI projects in the city identified an initial list of 35 GI professionals of whom the informant was aware or with whom the informant had actively collaborated; (2) the key informant contacted all of the professionals on the initial list via email, e-introducing them to members of the research team; and (3) a member of the research team then emailed the 35 professionals to request their participation in the study and to identify other GI professionals who they believed were part of the network and should also be included in the study.

Our non-probability, snowball sample was intended to augment the initial list provided by the key informant and produce as complete a list of members of our population as possible [117]. Recommendations from those initially identified resulted in a list of 16 additional potential members of the population. Of these 16, 11 did not respond to any of our attempts to contact them, and five declined to participate because they saw themselves as minimally involved in GI projects and not viable candidates for the study. Our key informant reviewed the list of potential additions and recommended that none of the 16 were involved enough in GI projects to justify meaningful inclusion in the sample. Thus, our final population of GI professionals for survey deployment was 35. Of these 35, two declined to participate because of their supervisory or regulatory role in the region, but they both agreed to allow their names to be listed on the survey roster (and thus as part of the network). We administered the survey to the remaining 33 potential respondents; 28 completed the survey, for a response rate of 80%.

### Survey instrument

Respondents completed an online survey generated using SurveyGizmo software [118], accessing the online survey by clicking on a link provided in an emailed invitation to participate; the survey was administered over the course of six weeks in early spring 2017. The survey assessed social network ties between GI professionals using a whole network roster method [119]. A roster with the names of 35 GI professionals, along with their titles, was presented to respondents and they were asked to check the boxes next to the names of people who satisfied the requirements of each question. The networks collected via these questions were arranged into adjacency matrices. In each matrix, a value in cell x_ij_ indicated that *i* nominated or rated *j* for a given relation, 0 indicated that *i* chose not to nominate *j*, and missing values indicated that *i* did not respond to the survey. We used UCINET VI, version 6.512 [120] to calculate the network measures, described below.

### Measures and analysis

#### Network measures

Past research using social network data collection in the field of environmental governance often characterizes communication or collaboration networks only. In addition to collecting information to construct a collaboration network, we measured frequency of collaboration, and collected information on trust networks based on two types of trust: *cognition-based trust*, in which a person trusts another to be competent and able at work-related tasks; and *affect-based trust*, in which a person trusts another to be respectful and act with goodwill and compassion [78,79] (Table 1). We combined these two networks by summing their matrices to create one trust network.

**Table 1:** Descriptions of network measures analyzed and survey questions used to collect necessary data.

Measure	Question	Description	Citation
Informal Leadership [*Dependent variable*]	Which people are most critical for achieving green stormwater infrastructure in Cleveland?	*In-degree*: count of the total number of nominations (in-coming ties) received by each individual.	[96,99]
Collaboration Network Size	Please check off the names of those people with whom you have worked regularly over the past [12 months] on green stormwater infrastructure projects.	*Degree*: count of the number of collaboration ties (incoming and outgoing) for each individual.	[121]
Collaboration Network Centrality	“”	*Betweenness*: measures the extent to which the focal individual acts as a bridge between otherwise unconnected others in the overall network.	[121,122]
Collaboration Network Openness	“”	*Effective size*: extent to which a focal individual is connected to people who are themselves not connected (within the immediate network around the individual).	[101,123]
Frequent Collaboration Network	How frequently do you interact with each person [in your collaboration network]? (1 = less than once per month; 5 = daily)	The network was dichotomized according to the rule: cell values greater than or equal to 3 (once per week, 2–3 times per week, daily). *Degree* thus measures collaboration ties that interact at least once per week.	[121]
Cognition-based Trust Network Size	I can rely on this person to complete tasks they agreed to do for me. (1 = strongly disagree; 7 = strongly agree)	Both networks dichotomized according to the rule: cell values greater than or equal to 5 (slightly agree, agree, strongly agree) were replaced with a 1. Cell values less than or equal to 4 (neutral, slightly disagree, disagree, strongly disagree) were replaced with a 0. Therefore, a 1 in cell *ij* indicates that *i* at least slightly agrees that they trust *j*. The two trust networks were moderately and significantly correlated (*r* = .47, *p* < .10). A combined trust network was created by summing the matrices of the two networks. Therefore a 1 in cell *ij* indicates that *i* feels one type of trust in *j*, while a 2 indicates that *i* feels both types of trust in *j*. *Degree*: sum of the total number of (incoming or outgoing) ties (of values of 1 or 2) for each individual.	[124,125]
Affect-based Trust Network Size	I feel comfortable going to this person to share problems and difficulties that I am facing. (1 = strongly disagree; 7 = strongly agree)		

#### Background and demographics

Gender. We used gender as a control and a moderator in our analyses because perceived leadership is often affected by gender [67,113]. Gender is a binary variable, coded so that 1 = female and 2 = male.

Organization type. Two members of the research team independently coded the respondents’ organizations into types. The resulting classifications were highly correlated (*r* = .82, *p* < .05). Differences were resolved in discussion. The resulting categories were: Community NGO, Environmental NGO/Land Trust, Federal Government, Local Government/Regional Authority, and University/Contractor.

Individual Role. In a similar manner, two research team members independently coded the respondent’s job titles into role types. The resulting classifications were highly correlated (*r* = .85, *p* < .05). Differences were resolved in discussion. The resulting roles were: Project Coordinator, Project Development, Project leader/Oversight, Researcher/Advisor.

## Results

### Network maps

**Fig 1:**
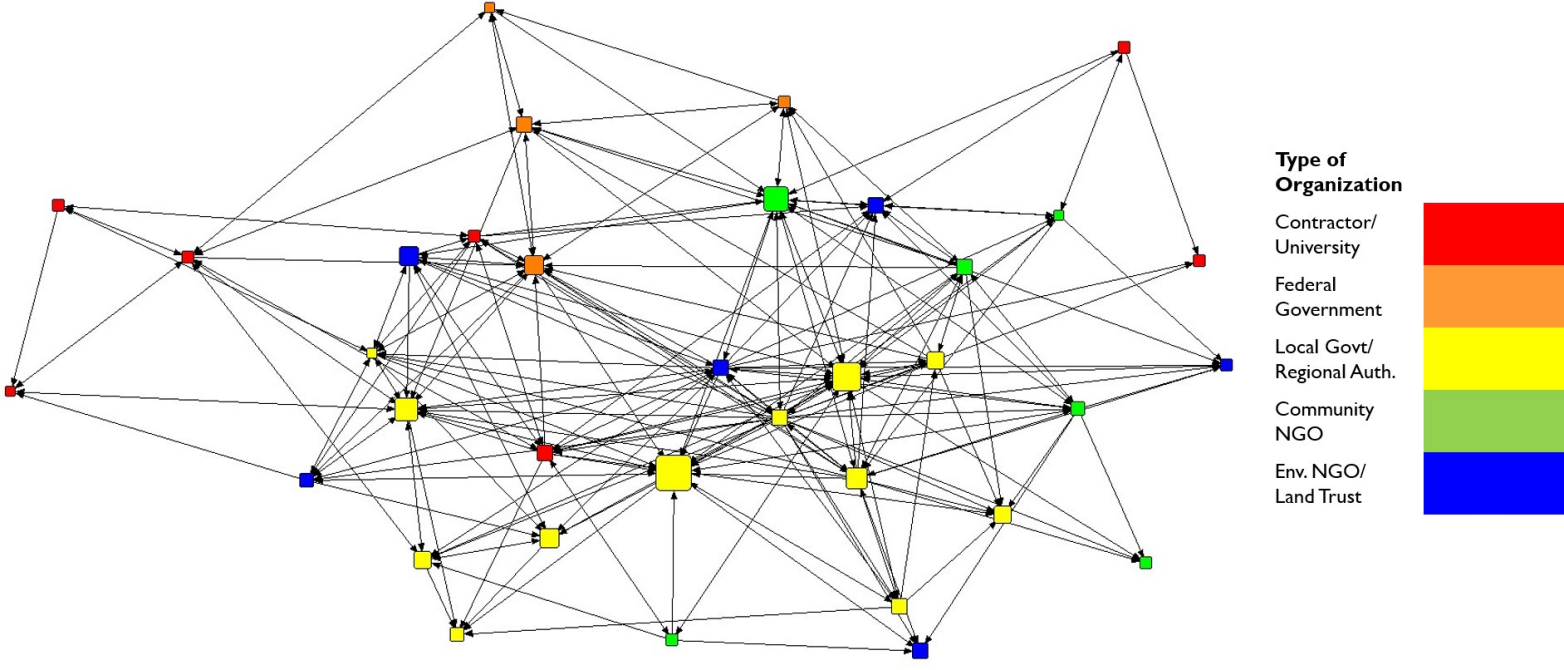
Collaboration network by organization type. Nodes are sized by number of informal leadership nominations received.

**Fig 2:**
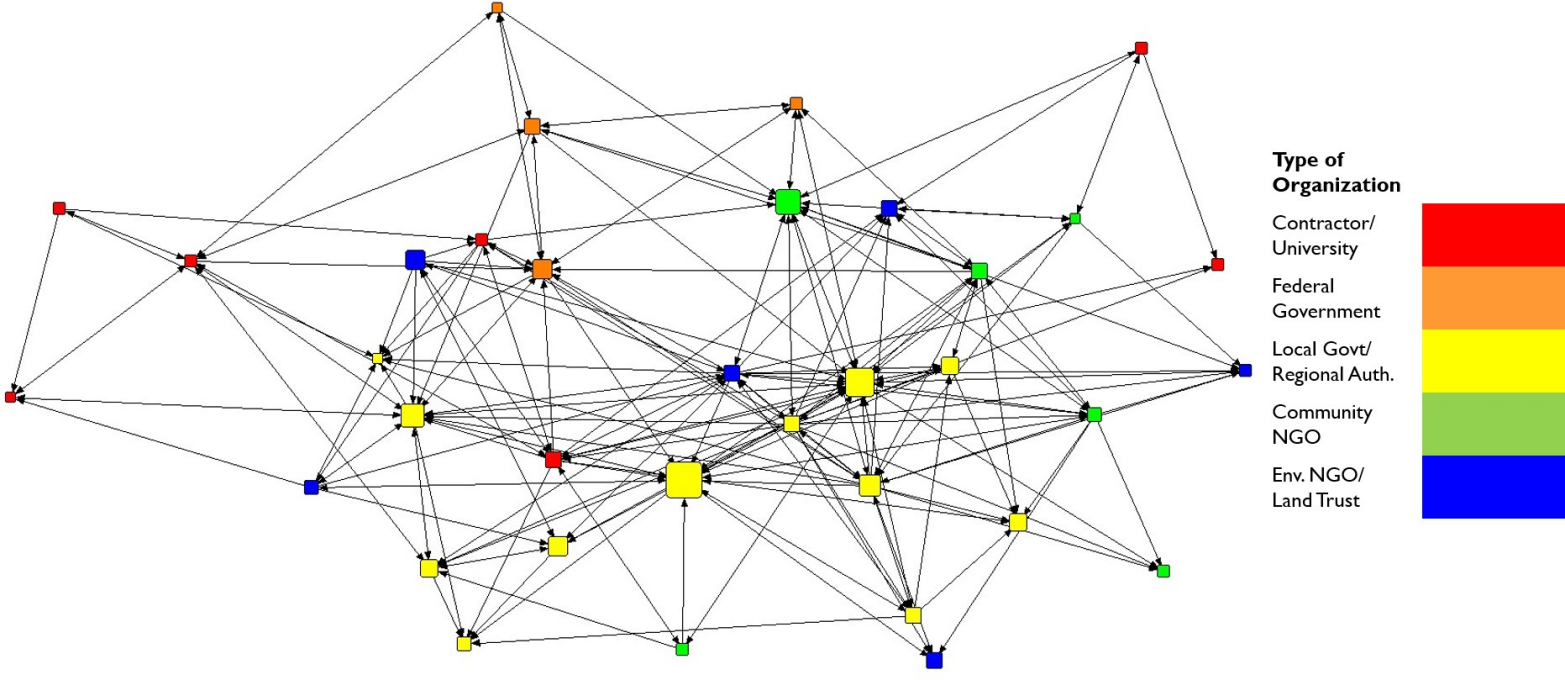
Trust network by organization type. Nodes are sized by number of informal leadership nominations received.

**Fig 3:**
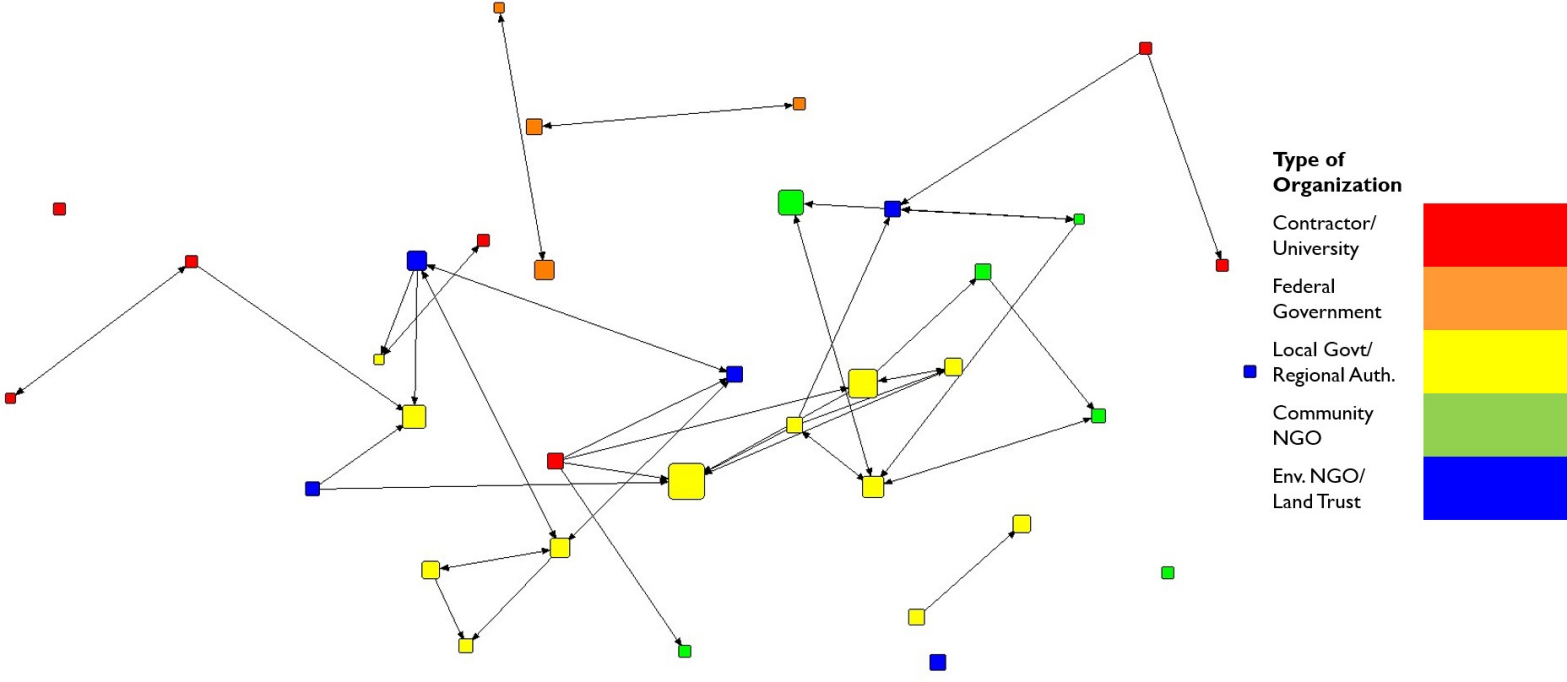
Frequent collaboration network by organization type. Ties indicate interaction at least once per week; nodes are sized by number of informal leadership nominations received.

Lines between nodes in the collaboration network visualization or map (Fig 1) indicate the presence of an informal relationship through which individuals (nodes) engage collaboratively in GI projects. Similarly, Fig 2 presents the trust network which depicts self-identified trust between individuals in the network. Although these two networks are highly correlated (QAP correlation 0.90), note that there are fewer trust relationships than collaboration relationships; some collaborations are marked by trust between individuals, while others are not. Due to the correlation between the two networks within this context, there were similar effects on leadership nominations, the dependent variable. Despite these similarities, we retain both the networks in our analysis herein to enhance future comparisons to studies of different contexts. Fig 3 demonstrates that most collaborators in the Cleveland GI network do not interact with high frequency.

### Frequencies and correlations

The 28 survey respondents did not differ significantly from non-respondents with regard to demographic variables such as gender, project role, and organization type (Table 2); the peer-nominated leadership is negatively and marginally significantly correlated with gender, such that women are more likely to be named as informal leaders (-.33, *p* < .10) (Table 3). Informal leadership is also positively and significantly correlated with collaboration network size (.62, *p* < .01), betweenness centrality (.47, *p* < .05), and openness (.58, *p* < .01), as well as with frequent collaboration network size (.59, *p* < .01) and trust network size (.66, *p* < .01), such that individuals with large collaboration and trust networks, frequent contact with others, and measurements of high betweenness centrality and openness in their collaboration networks are more likely to be identified as informal leaders (Table 3). There are no significant correlations between informal leadership and role or organization type.

**Table 2:** Frequencies for categorical variables.

Variable		Frequency	Percent
Gender			
	Female	14	50.0%
	Male	14	50.0%
Individual Role			
	Project Coordinator	3	10.7%
	Project Development	6	21.4%
	Project leader/Oversight	13	46.4%
	Researcher/Advisor	6	21.4%
Organization Type			
	Community NGO	5	17.9%
	Environmental NGO/Land Trust	3	10.7%
	Federal Government	4	14.3%
	Local Government/Regional Authority	10	35.7%
	University/Contractor	6	21.4%

**Table 3:** Means, standard deviations, and zero-order correlations.

Variable		*M*	*SD*	1	2	3	4	5	6	7	8
1	Informal Leadership	5.25	5.14								
2	Gender			-0.33†							
3	Role			-0.16	0.10						
4	Organization Type			0.26	-0.19	-0.18					
5	Collaboration Network Size	13.21	6.72	0.62**	-0.28	-0.19	0.24				
6	Collaboration Network Centrality	91.14	101.40	0.47*	-0.05	0.07	0.06	0.78**			
7	Collaboration Network Openness	9.42	5.76	0.58**	-0.25	-0.12	0.14	0.98**	0.86**		
8	Frequent Collaboration Network Size	2.83	2.44	0.59**	-0.32	-0.22	0.19	0.73**	0.69**	0.76**	
9	Trust Network Size	10.24	11.56	0.66**	-0.25	-0.18	0.17	0.93**	0.86**	0.95**	0.78**

*Note*. Table presents bivariate correlations.

† *p* < .10.

* *p* < .05.

** *p* < .01

### Analyses used to examine research questions

To examine our central research question, we used network analysis to create variables that describe individuals’ network properties (also called node-level network variables), including network size (e.g., degree centrality), position (e.g., betweenness centrality), openness (e.g., efficiency), and frequency of communication. Node-level network variables can be used in common statistical tests such as Pearson correlations, OLS regressions, logistic regressions, and others, as long as they meet the assumptions of the statistical tests being used. Because node-level network variables can sometimes have autocorrelation issues, we ran Durbin Watson tests on all models, and compared the results to dL and dU values based on our sample size and number of regressors [126]. We found no indications of autocorrelation, indicating that our data satisfied the independence of observations assumption.

Because our outcome variable is a count variable (number of times each person was nominated as an informal leader), we used Poisson loglinear regression model, which is used to predict a dependent variable that consists of count data. A comparison of the mean (4.80) and standard deviation (4.73) of the outcome variable indicated a Poisson distribution with equidispersion.

As is often found in network analysis, the network variables are highly correlated. Due to the high correlations, and resulting multicollinearity issues, it was not possible to include all network variables in one model. Therefore, we entered each network variable in a separate model and compared the relative effects of each one on the dependent variable of informal leadership using effect size and goodness of fit comparisons in Poisson loglinear regression models (S1 Table). Comparisons of Pearson χ^2^, log likelihood and likelihood ratio χ^2^ determined which model obtained the best fit with our sample data. Model 1 presents results of the individual characteristics predictor variables: among GI professionals in our study, women (*χ*^*2*^ = 8.47, *p* < .01) receive 1.8 times more leadership nominations from their peers, and employees of local government or regional authorities (*χ*^*2*^ = 5.62, *p* < .05) receive 1.9 times more.

In Model 2, we added collaboration network size. In the presence of the new variable, gender remains significantly related to informal leadership (*χ*^*2*^ = 6.03, *p* < .05). Employees of local government are no longer significantly related (*χ*^*2*^ = 1.90, *ns*). Collaboration network size is significantly and positively related to informal leadership (*χ*^*2*^ = 6.78, *p* < .01). In Model 3, we added collaboration network position (betweenness centrality). It is significantly and positively related to informal leadership (*χ*^*2*^ = 8.62, *p* < .001). In the presence of collaboration network position, women remain significantly more likely to be nominated as informal leaders (*χ*^*2*^ = 10.42, *p* < .001). In Model 4, collaboration network openness (effective size) is significantly and positively related to informal leadership (*χ*^*2*^ = 5.94, p < .05). In the presence of collaboration network openness, women remain significantly more likely to be nominated as informal leaders (*χ*^*2*^ = 6.37, *p* < .05). In Model 5, frequent collaboration is significantly and positively related to informal leadership (*χ*^*2*^ = 4.02, p < .05). In the presence of frequent collaboration, women remain significantly more likely to be nominated as informal leaders (*χ*^*2*^ = 5.21, *p* < .05). In Model 6, trust network size is significantly and positively related to informal leadership (*χ*^*2*^ = 14.61, p < .001). In the presence of trust network size, women remain significantly more likely to be nominated as informal leaders (*χ*^*2*^ = 7.79, *p* < .01).

Since all the network variables are significant predictors of informal leadership, effect size and goodness of fit comparison helps to interpret which network variables are the most important predictors. Effect size comparison reveals that every one unit increase in collaboration network size increases informal leadership nominations 1.04 times, every one unit increase in trust network size increases informal leadership nominations 1.03 times, and every one unit increase in frequent collaboration increases informal leadership nominations 1.08 times. Goodness of fit comparison reveals that the models including collaboration network size, frequent collaboration, and trust network size have the best fit with the data. Based on these comparisons, it appears that collaboration network size, frequent collaboration, and trust network size are the most important predictors of informal leadership. In addition, in all models, women are significantly more likely to be nominated as informal leaders.

**Fig 4:**
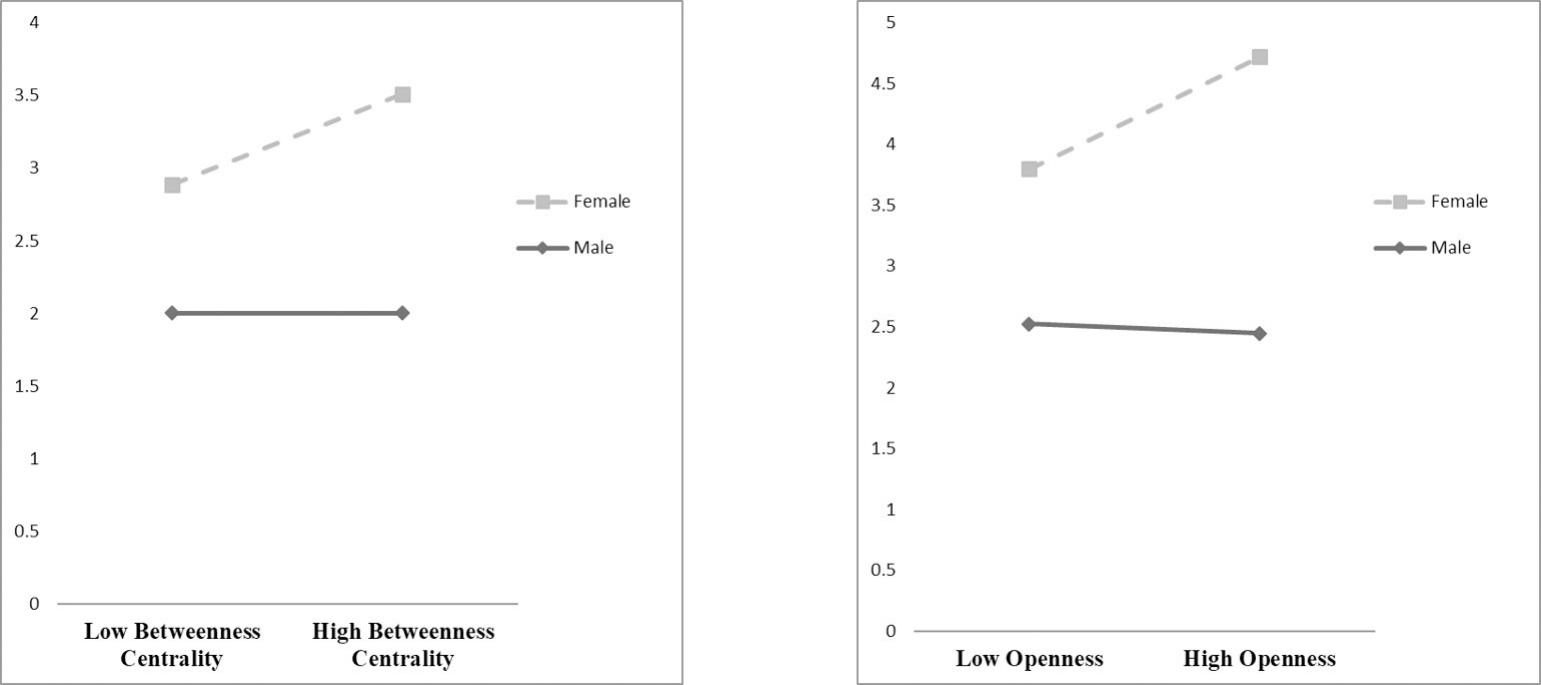
Effect of interaction between leadership nominations and network centrality on informal leadership nominations.

A secondary goal of the study was to examine potential differences between our GI implementation context and the business context within which most research on informal leadership emergence in networks has been done. Given the comparatively smaller effects of betweenness centrality and individual network openness on leadership nominations, we conducted an ad-hoc analysis to explore the potential moderating effect of gender on these variables. Past research in for-profit firms tends to show that men benefit more from brokerage positions than women [71,73,127,128]. Using Poisson loglinear regression models, we tested the interaction between betweenness centrality and gender (*χ*^*2*^ = 5.59; *p* < .05) and the interaction between openness and gender (*χ*^*2*^ = 10.49; p < .01) (S2 Table). Both interactions significantly predicted leadership nominations. Interestingly, contrary to previous research, brokerage positions (betweenness and openness) were beneficial for women, but not for men (Fig 4).

## Discussion

In this research, we aimed to better understand leadership in an informal network of GI professionals by measuring ‘ascribed leadership’—nominations of individuals as “critical for achieving green stormwater infrastructure in Cleveland.” Our results indicated that 14 out of 35 individuals in the GI professional network were in the 50^th^ percentile of leadership nominations, and 5 individuals were in the 75^th^ percentile of nominations. Informal leaders identified were: (1) more likely to be female (10 of 14); (2) more likely to have larger collaboration and trust networks; and (3) more likely to collaborate frequently (at least once per week) (S1 Table). Additional analysis showed that women who occupied brokerage positions within the network (betweenness centrality and openness) were more likely to be nominated by their peers as informal leaders (*see* S2 Table and Fig 4).

### Perceptions of leadership and the role of leaders in environmental governance networks

Leadership is generally ascribed to individuals for two reasons: (1) a perceived ‘match’ between an individual and a ‘leader prototype’ envisioned by the potential follower (implicit leadership theory [57,67,113]); and (2) perceived effectiveness of an individual [54]. Leader prototype ascriptions tend to favor men, both because of gendered legacies in perceptions of leadership, and because of a perceived correlation between leader prototype of those who already hold formal positions of leadership within organizations—also historically dominated by men as a result of a tangled web of structural inequalities in both organizational governance and society writ large. Perceived effectiveness ascriptions tend to favor people who are skilled at managing social interactions such as collaboration; these leadership nominations likely favor people who demonstrate social prowess, successful outcomes such as accomplished goals or projects, and/or a depth of knowledge or skills relevant to the goals of the network [54]. Effectiveness (and thus nominations for informal leaders) may also be enhanced by a central position within the network which provides: (1) information benefits, i.e. faster access to more and diverse information; (2) control benefits, i.e. the opportunity to control the flow of information through the network and the opportunity to connect people who would benefit from the new connection [103,104,129]; and (3) bricolage benefits, i.e. out of necessity, individuals learn to translate between different parts of the network and in the process, gain skills in understanding, interpreting, and translating diverse viewpoints and areas of expertise, which in turn makes the central actor a stronger, more creative exchange partner [102].

That women are more likely to be nominated than men as informal leaders is not unique (see Sec 2.4), but it is particularly interesting in the context of stormwater GI in Cleveland. Of the top 14 informal leaders identified in this study (10 female/4 male), all 4 males occupied high-level positions of formal leadership within their respective organizations (e.g. senior positions in local, regional, or federal government), while the 10 female leaders were split between positions of senior leadership and mid-level project coordination, development, or specific project management within their organizations. Nominations for male leaders can likely be explained by the implicit leadership theory (leadership prototype association) as well as by perceived effectiveness of those nominated, which we can assume given the individuals’ rise to positions of formal leadership within their respective organizations. In addition, the 4 male individuals nominated as leaders represent organizations with substantial influence over GI implementation in Cleveland generally; these organizations hold most of the financial, logistical, and political capacity historically leveraged to implement stormwater management in U.S. cities, like Cleveland. What’s unique here is that perceptions of a prototypical leader (including organizational affiliation relevant to this context) do not adequately explain the significant likelihood of women identified as informal leaders.

We hypothesize that in this context—a relatively new, mixed approach to GI for stormwater management combining large, centralized projects with smaller, decentralized efforts (and plagued with scientific, financial, and social acceptance uncertainties)—collaborative leadership behaviors (e.g. connecting individuals who would not normally work together, working collaboratively through anticipated problems of design, engineering, and/or implementation) may be more valued and recognized as leadership behaviors than are behaviors associated with more top-down approaches to managing team completion of tasks. This resonates with previous findings that women are more likely to serve as informal leaders in situations necessitating navigation of complex social relations [54,113]. Related to the more diverse roles held by women leaders within their respective organizations, it is possible that in this context women tend to be the actors with the most on-the-ground knowledge, access, and thus capacity to ‘get things done’ related to GI projects, which corresponds neatly with previous definitions of network leadership [96,99,100]. This is supported by a qualitative assessment of the reasons cited as to why individuals in the network “desire more collaboration” with the top 10 nominated female leaders—the most cited explanation (29% of total) given was that these informal leaders possess general expertise in the technical aspects of GI (science and engineering). Network participants also desired more collaboration with these female leaders because they represent potential support or funding sources (10%), respondents saw female leaders as legitimate or critical to building legitimacy in GI projects (5%), and female leaders represented opportunities to expand GI and associated co-benefits of projects, such as increased pollinator habitat or urban greening (5%). Thus, our identification of female leaders in this stormwater governance context may be less about gender and more about the individual women in this network occupying strategic network positions and possessing the knowledge, skills, and/or access to capital that increases their capacity to support GI and contribute meaningfully to the success of projects (likely across all stages of GI implementation including planning, design, construction, maintenance, and monitoring).

### Leadership and network position

Although informal leadership is historically over-awarded to men, several factors have been found in research to mitigate or reverse this relationship, including group gender composition, group personality composition, individual leader personality traits, and network centrality or multiplexity. Group gender composition does not play a role in this research context, given the equal split between men and women in the network, and we did not collect data to investigate group personality composition or individual personality traits; in addition, this latter category may play less of a role in multi-organizational, large-scale, environmental issue-oriented networks.

Our results do suggest, however, significant interactions between gender and network brokerage (as measured by betweenness centrality and openness) in predicting leadership nominations (Fig 4). Betweenness describes the extent to which an individual directly connects otherwise unconnected actors in a network (e.g. as a bridge between people) [98], while openness describes the extent to which an individual is connected to actors who are themselves not connected within the immediate network around the individual [129]. Together, both of these measures describe the brokerage role of an individual—a position of influence within a network structure relating to access and distribution of information and other capitals [102]. Women nominated as leaders in the Cleveland GI network benefit from occupying these central brokerage roles (Fig 4); in past research these positions have tended to favor men [71,73,127,128].

One interesting phenomenon demonstrated in our data is that collaboration network betweenness centrality and openness are highly correlated with trust network size. Simmel [130] suggested that strong ties, such as trust ties, are more likely to exhibit closure than weak ties, a property he referred to as Strong Tie Triadic Closure Property. Granovetter [131] used this property in developing his “strength of weak ties” theory that explained why weak ties are more likely to provide non-redundant information than strong ties, because weak ties are more likely to exist in open, rather than closed, triads. However, Burt [129] argued for a differentiation between the properties of the network structure and the properties of the network tie, stating that the benefits that come through spanning structural holes are better understood as deriving from the network structure than from the strength or weakness of the tie. Our data supports Burt’s view, in that it shows that it is not impossible for an individual to have many strong ties while also having a network characterized by openness. An open network structure and strong network ties are not necessarily mutually exclusive.

In the context of GI for stormwater management, which is unlike much previous research on network leadership—in that it includes a diversity of approaches and organizations, a historical legacy of cultural associations with stormwater management, and a host of barriers such as scientific uncertainty—the effectiveness of an individual appears to be more important than whether they fit a leadership prototype. Effectiveness is theoretically enhanced by occupying brokerage roles, and this helps further explain why our results differ from previous research. Our results may also have been influenced by word choice within our survey instrument. We purposefully avoided using the word “leader” in survey questions and instead asked respondents to identify individuals who were “critical” to achieving GI results and successes in Cleveland. Rather than triggering a leader prototype response, we aimed to collect information on individuals who were essential to, and thus leaders in promoting GI across all stages of GI implementation. We made a conscious methodological choice not to use the word “leader” in an attempt to avoid personal biases associated with a leadership prototype, and as a result, we identified the potentially greater role that network position plays fostering the leaders in informal environmental governance networks and in amplifying the critical skills and competencies of these emerging leaders.

## Conclusion

This research attempts to expand our understanding of the relationship between network properties and leaders in informal environmental governance networks. In so doing, we describe specific associations of individual leaders with the contexts of a transition toward implementing GI for governing stormwater in an urban setting. In the majority of previous research on informal leadership in closed organizations, a leadership prototype perceived by followers has often explained nominated leaders; in our study, we found that network size, frequency of collaboration, and network positions of brokerage are more useful in defining peer-nominated leaders in these informal networks. Specifically, in the context of stormwater governance in Cleveland, OH, women were recognized as leaders more significantly than men, controlling for all other factors such as formal position held and organization represented. In addition, women who occupied brokerage positions within the network were more likely to be identified as leaders, while men did not benefit in the same way from brokerage positions. Although this finding in itself is interesting, it clearly illuminates that factors beyond perceived leadership prototype determine leadership in informal environmental governance networks that coalesce toward a specific social-ecological goal. Our findings suggest that network size, frequency of collaboration, and position, particularly centrality and openness, combined with individual competencies and knowledge play a critical role in the identification of network leaders.

Our research findings have limitations. For instance, because of the cross-sectional nature of our data, we cannot make causal direction assumptions regarding all relationships. It is possible that individuals who are seen as leaders by others in the network are able to attain better network positions by virtue of their leadership. Additionally, a single case study cannot be generalized across all contexts. Thus, additional research is required in different types of environmental governance contexts to determine factors linked to informal network leadership both in common with and disparate from our GI stormwater governance network.

These empirical findings also highlight the need for additional research. Specifically, questions arise such as to what degree do the contexts of a particular environmental governance network interact with the leaders that arise in the network? Do a set of contexts impact the structure of the network; for example, do water governance networks interact with centrality differently than do wildlife management networks? Under certain environmental conditions, which actors (individuals or organizations represented) occupy bridging or positions of brokerage most commonly? Does this structure impact leadership generally, or a typology of leaders, specifically? Future research should also explicitly investigate the impact of network position on peer-nominated leaders in environmental governance networks. Studies should include both urban and rural social-ecological contexts and compare leaders’ network positions and traits across a variety of network structures and sizes. With regard to networks formed around GI for stormwater management, a logical next step would be to conduct similar studies in a number of cities implementing GI to assess how overall network properties might affect GI success through the multi-city measurement and comparison of metrics such as implemented projects, gallons of stormwater mitigated, or a similar measurement of the overall transition toward leveraging GI for stormwater management.

## Supporting information

S1 Table Results from poisson loglinear regression predicting informal leadership. (DOCX)

S2 Table Results from poisson loglinear regression predicting informal leadership and interactions between network measures and gender. (DOCX)

S1 File Anonymized response data from a network survey of green stormwater infrastructure professionals in Cleveland, OH. (XLSX)
