# Supplementary material for: Leadership in informal stormwater governance networks
Source: PLoS One. 2019 Oct 17;14(10):e0222434. doi: 10.1371/journal.pone.0222434 (PMC6797200; doi:10.1371/journal.pone.0222434)
Supplement: S1 Table — (DOCX) [file pone.0222434.s001.docx]

Supplementary Information for

**Leadership in informal stormwater governance networks**

Brian C. Chaffin*, University of Montana

Theresa M. Floyd, University of Montana

Sandra L. Albro, Holden Forests & Gardens

*Email: [brian.chaffin@umontana.edu](mailto:brian.chaffin@umontana.edu)

Table S1. Results from Poisson Loglinear Regression Predicting Informal Leadership.
